# A Nanobody Toolbox for Recognizing Distinct Epitopes on Cas9

**DOI:** 10.1016/j.jmb.2024.168836

**Published:** 2024-10-30

**Authors:** Jack Boylan, Rebecca A Shrem, Isabel C. Vallecillo-Viejo, Craig L. Duvall, Brian E. Wadzinski, Benjamin W. Spiller

**Affiliations:** 1 - **Departments of Pharmacology,** Vanderbilt University, Nashville, TN 37232, United States; 2 - **National Institutes of Health,** National Institute of Allergy and Infectious Diseases, Bethesda, MD 20892, United States; 3 - **Departments of Biomedical Engineering,** Vanderbilt University, Nashville, TN 37232, United States; 4 - **Departments of Pathology, Microbiology and Immunology**, Vanderbilt University, Nashville, TN 37232, United States

**Keywords:** nanobody, Cas9, Epitope bin

## Abstract

Cas9s and fusions of Cas9s have emerged as powerful tools for genetic manipulations. Fusions of Cas9 with other DNA editing enzymes have led to variants capable of single base editing and catalytically dead Cas9s have emerged as tools to specifically target desired regions of a genome. Here we describe the generation of a panel of nanobodies directed against three unique epitopes on Streptococcus pyogenes Cas9. The nanobodies were identified from a nanobody library derived from an alpaca that had been immunized with Cas9. The most potent binders recognize Cas9 and RNA bound Cas9 equally well and do not inhibit Cas9 cleavage of target DNA. These nanobodies bind non-overlapping epitopes as determined by ELISA based epitope binning experiments and mass photometry. We present the sequences of these clones and supporting biochemical data so the broader scientific community can access these reagents.

## Introduction

Since the first report of programmable DNA cleavage by CRISPR (clustered regularly interspaced short palindromic repeats)/Cas9 in 2012, this system has played a central role in new technologies that have enabled developments in the fields of genome engineering, epigenetics, and genome imaging. Some of these technologies have entered clinical trials and CASGEVY, a CRISPR/Cas9 gene editing therapy that treats sickle cell disease and transfusion dependent beta thalassemia, has already been approved.^1-4^

Chimeras of targeting and activating RNAs (single-guide RNA, sgRNA) have yielded a simple system (ribonucleoprotein complex of Cas9 and a single RNA) that creates double-stranded breaks at a targeted genomic site.^2,5^ Cas9 and catalytically-impaired Cas9 have been fused to deaminases, reverse transcriptases, and transposases to create variants capable of site-specific single base editing and insertions of DNA.^6-10^ This has resulted in a repertoire of enzymes that are readily available to the research community and routinely used to edit genomes in cells and organisms.

A limitation of Cas9 is low specificity and fidelity in the subsequent repair of double-stranded breaks by non-homologous end joining, which has been addressed by fusing catalytically dead Cas9 (dCas9) to other nucleases to enhance cleavage fidelity while maintaining the targeting fidelity of the guide RNA.^11^ Because dCas9 retains the ability to bind guide RNAs and to specifically bind DNA, it has been harnessed for applications emerging from the capability of Cas9-sgRNA to specifically localize to essentially any location in a genome. dCas9 has emerged as a tool of choice for genome imaging and sequence-specific protein delivery (e.g., transcription factors, epigenetic effectors, base editors, fluorescent proteins) to the genome.^12,13^ dCas9 is also used in CRISPR inhibition (CRISPRi) and CRISPR activation (CRSPRa). In these applications dCas9 with a guide can physically block transcription (CRISPRi) by preventing recruitment of the transcription apparatus, or in the case of CRISPRa, dCas9 can be fused to a transcriptional activator and enhance transcription.^14,15^ These approaches rely on the fusion of effector proteins to the amino or carboxy termini of Cas-9; however, the fusions are limited in the number of partners and specific three-dimensional geometry.

To expand possible strategies and geometries used to recruit potential partners to *Streptococcus pyogenes* Cas9 (SpCas9), we sought to develop nanobodies against SpCas9. These clones could be used to expand the ways to target enzymes to Cas9, or for other purposes. Nanobodies are small (12–14 kDa) antigen-binding proteins derived from the heavy-chain only antibodies found in camelids.^16^ Nanobody size, single-chain nature, and ability to retain function in eukaryotic cytoplasm^17^ suggests to us that they may be preferable to traditional antibodies as tools to target SpCas9, motivating this work. We anticipated that the small paratope size would reduce the likelihood that binders would inhibit SpCas9. We also posited that the single chain construction and retention of activity in cells would allow tagging and expression of these clones in cells. To accomplish our objective, we immunized an alpaca with purified SpCas9 and generated a library of V_HH_ genes encoding nanobodies from this animal. We subsequently identified clones from this library that bind tightly to Cas9, recognize distinct epitopes, bind to Cas9 when complexed with gRNA, and do not inhibit the activity of Cas9. Through these studies, we developed a set of anti-Cas9 nanobodies.

## Results

### Generation of anti-Cas9 nanobody library

**Figure 1.**
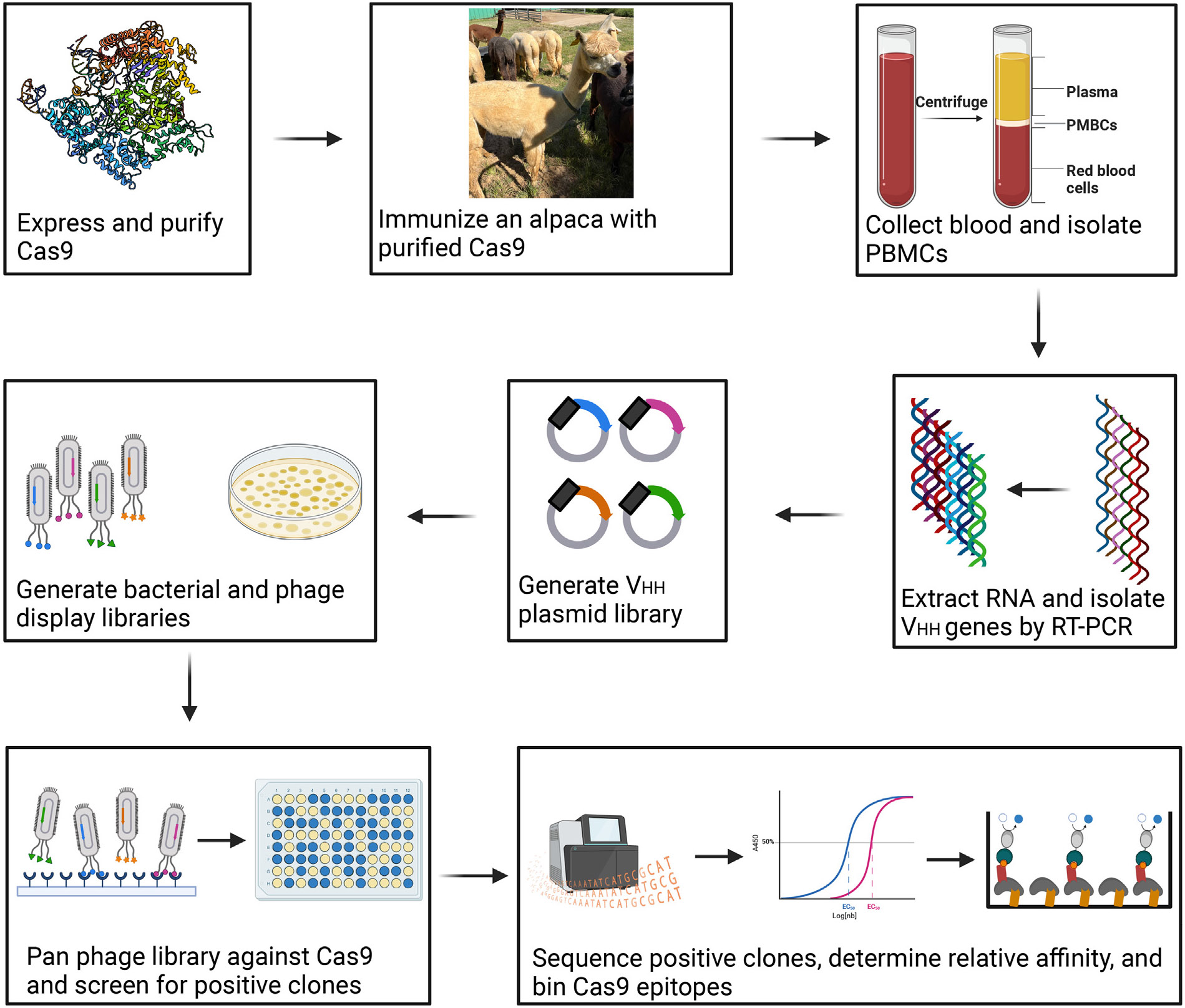
Summarized processes for generating and isolating alpaca-derived nanobodies against SpCas9.

As outlined in Figure 1 and described further in the Experimental Procedures, a single alpaca, “Flower Blossom,” was immunized with purified *Streptococcus pyogenes* SpCas9 to generate V_HH_ camelid single domain antibodies against SpCas9. Blood was drawn from “Flower Blossom” after the 8th immunization and peripheral blood mononuclear cells (PBMCs) were isolated. From the PBMCs, total RNA was purified, cDNA was reverse-transcribed, and V_HH_ phage display libraries were made. A single round of panning was performed against immobilized SpCas9 and 51 clones that recognized SpCas9 were identified.

### Sequence analysis and biochemical screens of nanobody library

All positive clones were sequenced and a multiple sequence alignment (MSA) from the corresponding protein sequences revealed that 39 of the 51 clones in the library possessed unique sequences (Supplemental Figure 1). The corresponding DNA sequences have been deposited in Genbank with accession numbers PP869893-PP869943. Further analysis using the IMGT database^18^ to map the likely unmutated common ancestor (UCA) origin of nanobodies in the library indicated that IGHV 3S66 was the inferred germline parent of 51% of the clones (Table 1). The presence of a dominant V-Gene in the library is consistent with other nanobody libraries generated from immunized alpacas.^19^ The overall gene usage of these nanobodies is constrained, with the entire library likely using only 5V, 5 D and 3J genes (Table 1).

**Figure 2.**
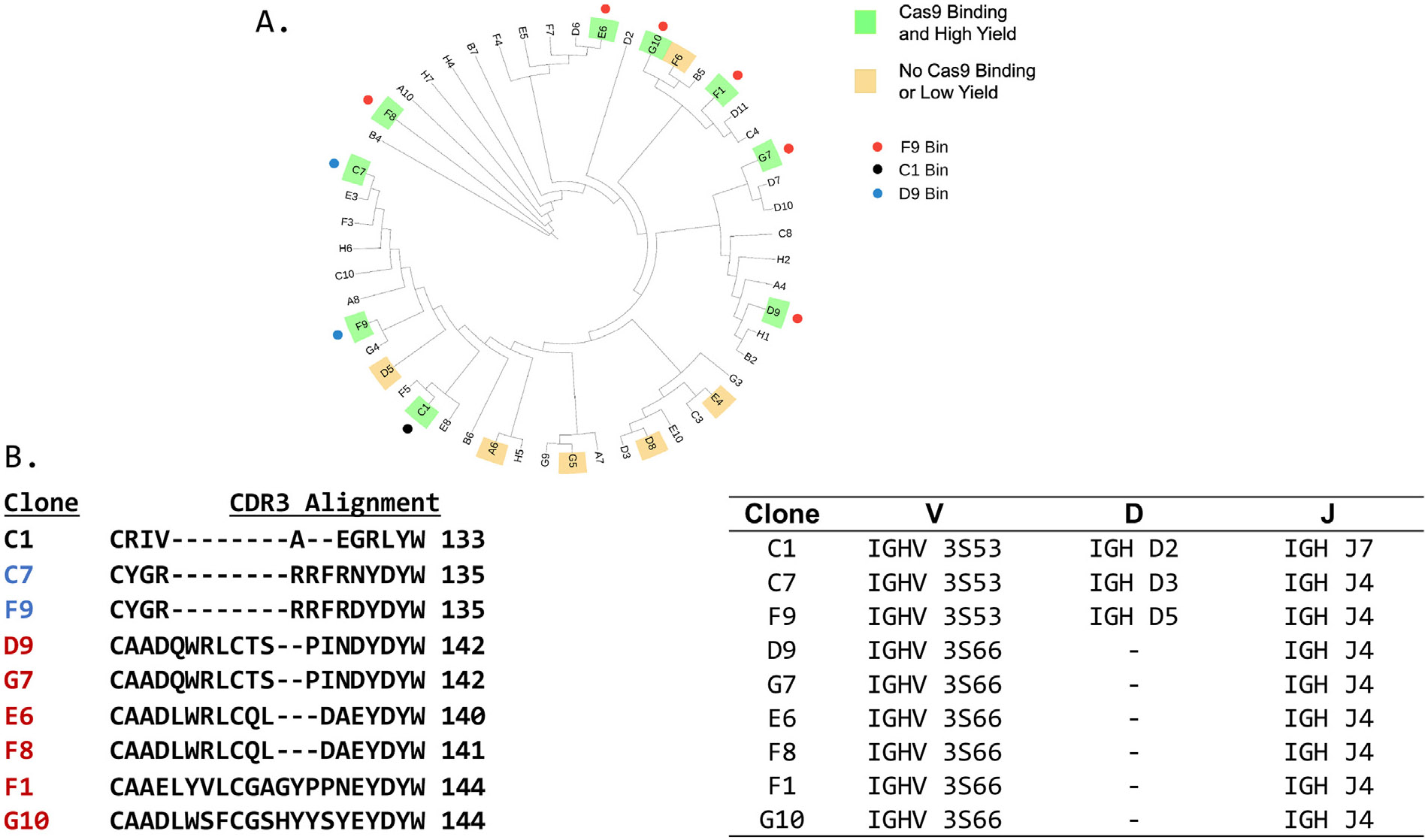
Summary of candidate clone selection and analysis. (A) Cladogram of the anti-Cas9 nanobody library. Selected candidate clones highlighted in green and yellow. Epitope binning results displayed. (B) Sequence alignment of the CDR3 regions of binned nanobodies. (C) Predicted V, D, and J gene usage for binned nanobodies.

A cladogram and identity matrix were used to select fifteen clones from the nanobody library (Figure 2A). We chose diverse sequences to increase the chance of identifying nanobodies that recognize multiple SpCas9 epitopes. We also focused on clones that could be chemically biotinylated for use in binding assays, and thus selected clones without lysine residues in the complementary determining region 3 (CDR3) region, which is commonly involved in antigen binding, and with >3 lysine residues outside of this region (Table 2), to facilitate biotin labeling with commercial N-Hydroxysuccinmide-biotin reagents in such a way that labeling would be unlikely to disrupt antigen binding. Biotinylated nanobodies were subsequently used in epitope binning experiments. The number of mutations and amino acid changes from the inferred UCA of each clone were calculated (Table 2). The number of mutations within this panel of clones ranged from 7 to 44 with an average of 20.8. The number of amino acid changes ranged from 3 to 23 with an average of 11.3.

**Figure 3.**
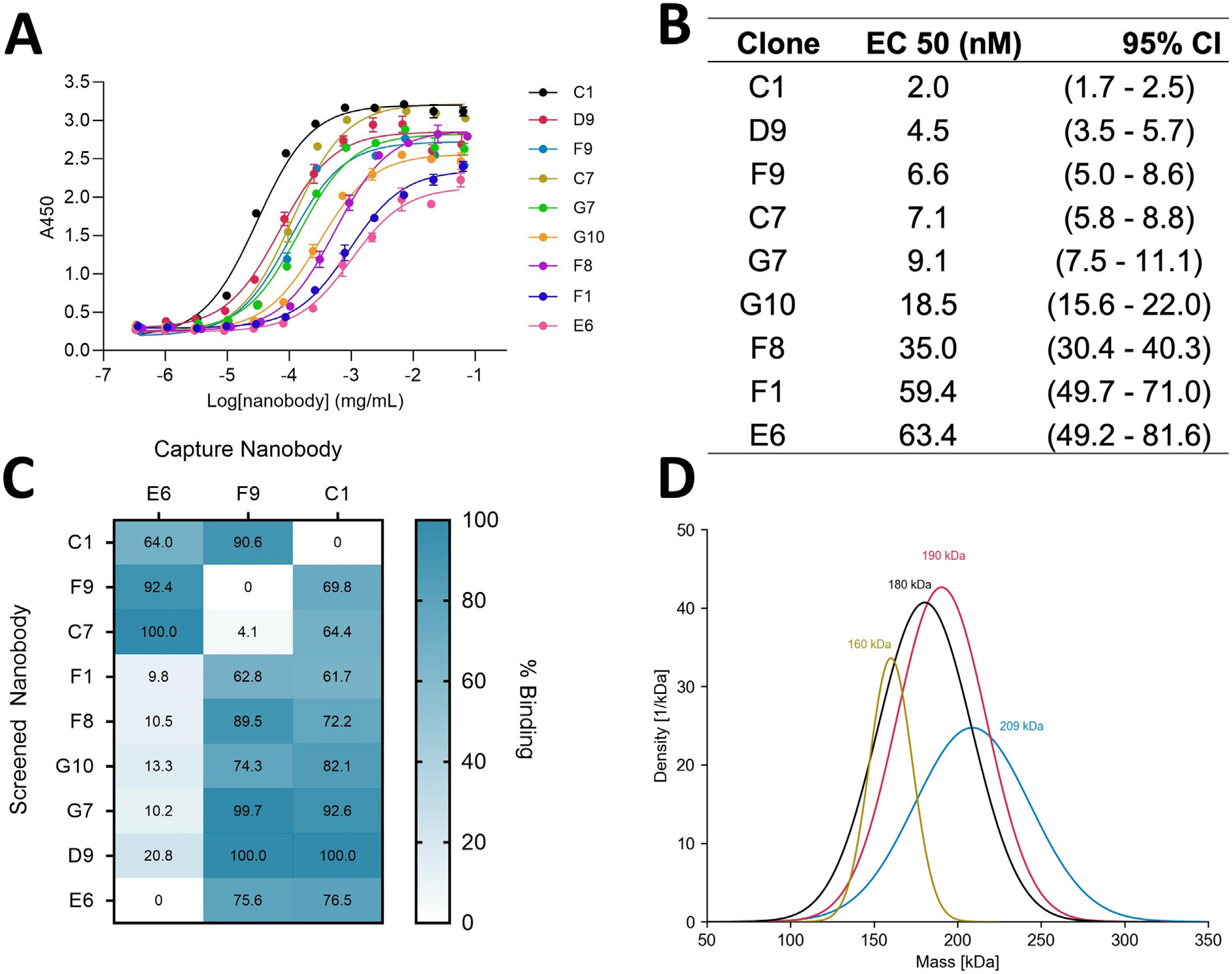
Analyzing and streamlining candidate clones. (A) Dose response curves of nine candidate clones to Cas9. (B) Half-maximal effective concentrations (EC50s) of these clones to Cas9 determined from non-linear regressions of the dose response curves. (C) Summary of epitope binning by sandwich ELISAs, in which Cas9 was captured with a single unbiotinylated nanobody and the percent binding for screened biotinylated nanobodies were obtained. (D) Confirming epitope bins with mass photometry by showing that the mass of Cas9 shifts 10–20 kDa when progressively incubated with nanobodies recognizing additional epitopes.

Clones D5, C7, F9, and C1 were expressed and purified from *E. coli*. Clones D9, E6, F6, F8, G7 D5, F1, and G10 did not express well in *E. coli*, and were purified from mammalian cells. Purified nanobodies were screened for binding to SpCas9 by enzyme-linked immunosorbent assay (ELISA) using a 12-point dilution series from ~80 μg/mL to ~4.5 × 10^−4^ μg/mL nanobody. As summarized on the cladogram (Figure 2), we obtained high yields (>2 mg) of a panel of nine clones that showed binding against SpCas9 (Figure 2A). For the remaining seven clones, SpCas9 binding was either not observed or protein could not be produced. The relative affinities of our panel of nine clones as measured by ELISA are shown in Figure 3. The dose responses for the selected nanobodies to SpCas9 showed a broad range of affinities and fitting these curves by non-linear regression allowed us to determine the half-maximal effective concentration (EC50) of the nanobodies against SpCas9. The clones all had nanomolar EC50s, ranging from 2 to 63.4 nM. Nanobodies C1, D9, F9, C7, and G7 bound the tightest to SpCas9, and exhibited relative affinities <10 nM (Figure 3B).

### Epitope binning

With a goal in mind of identifying clones that recognize non-overlapping sites on SpCas9, we used a sandwich ELISA to determine competition groups/bins of these nanobodies. In these experiments, SpCas9 was captured by a single unbiotinylated nanobody and dose–response curves for all nine biotinylated nanobodies against SpCas9 were obtained. For each unbiotinylated nanobody used to capture SpCas9, the area under the dose–response curve for the corresponding biotinylated nanobody was subtracted from the areas under the dose–response curves for all biotinylated nanobodies. The remaining area under the dose–response curve for each biotinylated nanobody was taken as a percentage of the area under the dose–response curve for the tightest binding biotinylated nanobody to quantify binding.

Capturing SpCas9 with clone E6 resulted in low levels of binding (<25%) for clones F1, F8, G10, G7, and D9, suggesting they recognize the same epitope on SpCas9 as does E6, whereas clones C1, F9, and C7 had high levels of binding (>60%), indicating they recognize a different epitope than E6 (Figure 3C). A second sandwich ELISA was performed using unbiotinylated F9 to capture SpCas9. High levels of binding (>60%) by nanobodies F1, F8, G10, G7, D9, and E6 were observed in the presence of unbiotinylated F9, suggesting these clones recognize the same SpCas9 epitope and bind a different epitope than F9 (Figure 3C). Low levels of binding (4.1%) were observed by C7 when SpCas9 was captured by F9, suggesting this clone recognizes the same SpCas9 epitope as F9 (Figure 3C). High levels of binding (90.6%) were observed by C1 when SpCas9 was captured by F9, suggesting this clone recognizes a different epitope than F9 (Figure 3C). A third sandwich ELISA was performed to verify that C1 recognizes a different epitope than the clones in the F9 and E6 bins. High levels of binding (>60%) by all clones were observed when SpCas9 was captured by C1, suggesting this clone recognizes a different SpCas9 epitope than the other clones in the panel (Figure 3C). To summarize, epitope binning resulted in the identification of three bins of nanobodies that each recognize a distinct SpCas9 epitope. The bins to which each clone belongs are indicated on the cladogram (Figure 2A). Alignment of the CDR3s of the binned clones reveals that C7 and F9 differ by a single amino acid in CDR3 and likely bind SpCas9 similarly (Figure 2B). Within the D9 containing clade four unique CDR3 sequences are evident. D9 and G7 are identical, with very similar EC50's (Figure 3B), E6 and F8 are identical in CDR3 although there are 5 differences in or near CDR2 (Supplemental Figure 1) that must be responsible for the ~2-fold difference in EC50 (Figure 3B). Clones F1 and G10 differ in 12 positions (9 within CDR3) and have ~3-fold different EC50's (Figure 3B and Supplemental Figure 1). All clones in bin 3 use the 3S66 variable region and J region J4. IMGT^18^ was unable to identify a D region for these clones (Figure 2C).

Mass Photometry (MP), a technique that uses light scattering to measure the mass of proteins was used to verify that the tightest binders in each bin could recognize SpCas9 simultaneously. Clones D9, F9, and C1 were selected for this experiment because they had the lowest EC50s in each bin. When SpCas9 was incubated with each additional nanobody, the average mass of the complex increased by 10–20 kDa (Figure 3D). SpCas9 alone showed an average mass of 160 kDa, which rose to 180 kDa in the presence of C1, then to 190 kDa with C1 and D9, and to 209 kDa with C1, D9, and 9 (Figure 3D). The ability of all three of these clones to bind SpCas9 simultaneously confirms that we have identified three bins of nanobodies that recognize distinct sites on SpCas9.

### Characterization of ribonucleoprotein complex (RNP)-nanobody interactions

Because SpCas9 must be complexed with a guide RNA (gRNA) to target the nuclease to a DNA sequence, we wanted to determine if the clones we’ve identified can bind SpCas9 when complexed with gRNA. We focused on C1, D9, and F9 because they are the tightest binders in each bin and determined their relative affinities for apo SpCas9 and an active SpCas9 RNP.

**Figure 4.**
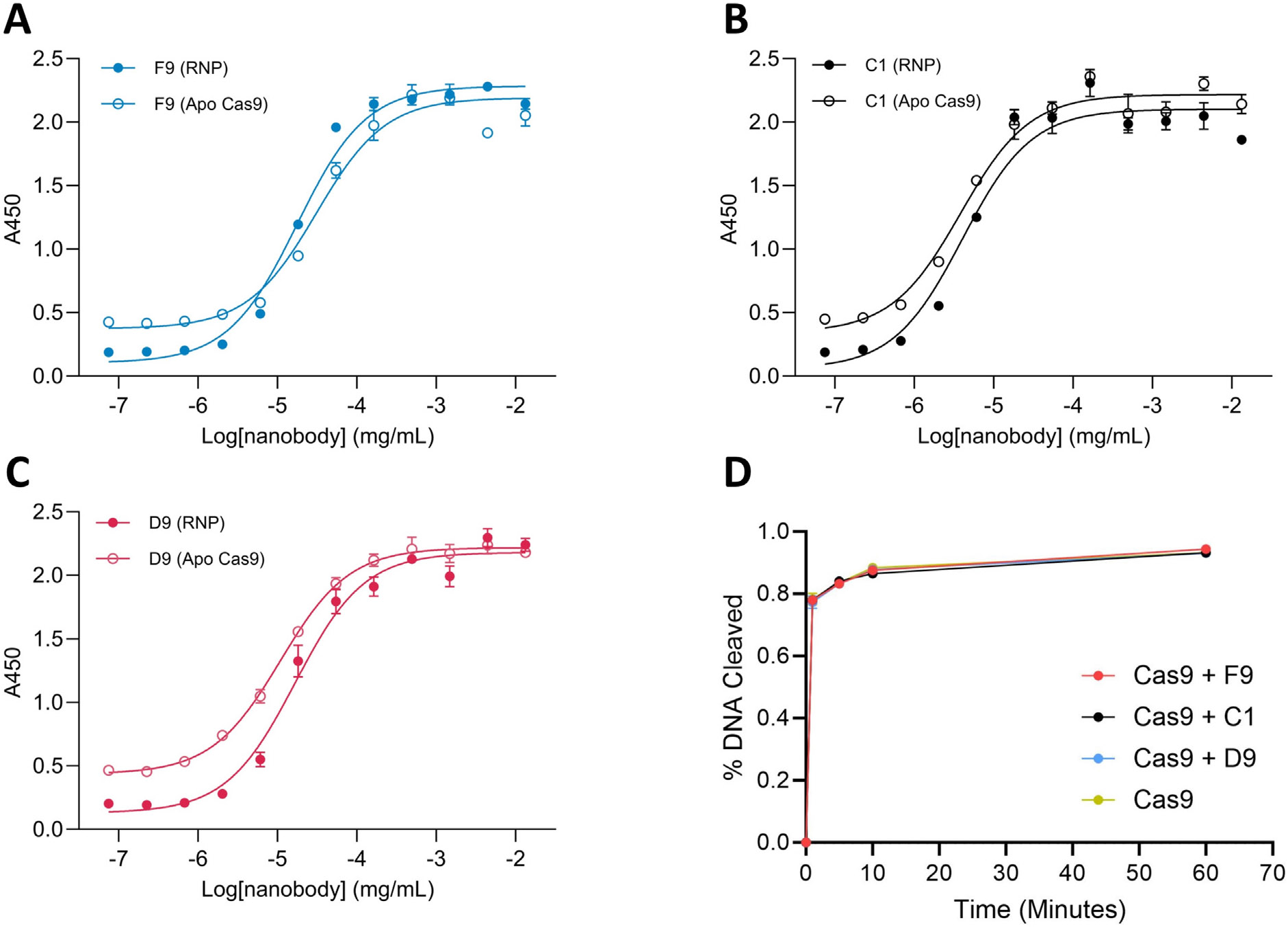
Interactions between tightest binders of each epitope and RNP. (A) Dose response of F9 to apo Cas9 and RNP. (B) Dose response of C1 to apo Cas9 and RNP. (C) Dose response of D9 to apo Cas9 and RNP. (D) Cas9 inhibition assay showing percent linearized plasmid cleaved over time by Cas9 alone and Cas9 incubated with clones F9, C1, and D9. Error bars are SD of triplicate measurements.

The active RNP was formed using purified SpCas9 and a gRNA targeted to the ROSA mT/mG plasmid.^20^ The dose–response of clones D9, F9, and C1 to this RNP and apo SpCas9 were obtained by ELISA. Non-linear regressions of these data demonstrate that these clones have similar relative affinities for the RNP and apo SpCas9 *in vitro* (Figure 4A-C).

The activity of Cas9 is required for CRISPR-Cas9 applications involving the generation of single or double-stranded breaks in the target DNA. To ensure our nanobodies can bind to the SpCas9 RNP without inhibiting its activity, we assayed the inhibitory effects of clones D9, C1, and F9 in a DNA cleavage assay. Each nanobody was incubated with the RNP complexed with the ROSA mT/mG gRNA before being added to linearized ROSA mT/mG plasmid DNA and percent DNA cleavage was assayed. The results show plasmid cleavage was equally prominent in SpCas9 incubated with each nanobody compared to parent SpCas9 alone (Figure 4D and Supplemental Figure 2), indicating our clones recognize RNP without inhibiting SpCas9 activity *in vitro*.

## Discussion

In this study, we immunized an alpaca with full-length SpCas9, generated a library of anti-SpCas9 nanobodies, and identified 51 clones reactive against SpCas9. IGHV 3S53, which is known to be a commonly used gene in the alpaca immune response,^19,21^ was the inferred unmutated common ancestor (UCA) of 23.5% of the clones (Table 1). From within our initial panel of 51 clones, we identified 9 that can be purified in multiple milligram quantities and bind tightly to SpCas9 (Figure 2A). The EC50's for these clones range from 2 to 63.4 nM with three of the four most potent binders having heavily mutated variable genes. Cones C1, F9, and C7 are potent binders with EC50's of 2, 6.6 and 7.1 nM and these clones have 20, 20, and 21 amino acid changes, respectively, from the likely UCA. D9 is also a potent binder with an EC50 of 4.5 nM despite having only 14 mutations and 7 amino acid changes. G7 binds nearly as tightly as D9 (9.1 vs 4.7 nM EC50) and is also lightly mutated. These clones have identical CDR3 regions and are on the same clade, suggesting that they arise from a particularly effective gene rearrangement that did not require heavy mutation to achieve tight binding (Figure 3B; Table 2). These clones use the IGHV 3S66 and IGH J4 V and J regions. These findings suggests that elevated levels of B-cell activation during the generation of V_HH_ genes correlate with higher affinity for clones C1, F9, and C7, and a particularly effective gene rearrangement is responsible for the potency of D9 and G7. The remaining clones (G10, F8, F1, and E6) have between 3 and 6 amino acid changes from inferred UCA and EC50s ranging from 18.5 to 63.4 nM.

Epitope binning allowed us to determine that these nine positive clones recognize three distinct sites on SpCas9 (Figure 3C). The cladogram of the nanobody library shows that clones recognizing the same epitopes have sequences with higher similarity than clones recognizing different epitopes (Figure 2A). The high levels similarity in the CDR3 regions of clones within the same bins also suggests that this sequence is involved in SpCas9 recognition and specificity (Figure 2B).

We confirmed that the tightest binders in each of our epitope bins can recognize SpCas9 simultaneously, and that these clones can bind to the active SpCas9 RNP without inhibiting its activity *in vitro* (Figure 4). This process resulted in the development of a toolbox of three anti-SpCas9 nanobodies (C1, F9, and D9) that bind tightly to distinct sites at SpCas9 and can likely be used for applications requiring enzymatic activity. We also verified that C1, F9, and D9 did not recognize Cas3, Cas12, or Cas14 (Supplemental Figure 3).

The ability of nanobodies to be readily fused to other proteins without affecting antigen recognition means that these clones can potentially be used to recruit histone and base modifying enzymes to target sequences with dead SpCas9 (dCas9) and Cas9 “nickases.” Previous efforts to use Cas9 to localize histone and base modifying proteins to target sequences with Cas9 have exclusively involved fusing or recruiting these enzymes to the N or C-termini of Cas9.^6,22-24^ Because our spCas9 nanobodies recognize three distinct epitopes, they may be useful for attaching genome modifying enzymes at sites other than the N and C termini on Cas9 and potentially expand the existing repertoire of tools for making targeted base and epigenetic modifications. Our nanobodies also have the potential to be used for other applications involving the recruitment of a target protein or oligonucleotide to Cas9, such as detecting Cas9 by proximity ligation, targeting Cas9 to specific cell surface receptors, and fluorescently imaging Cas9.

## Experimental Procedures

### Generating an anti-SpCas9 nanobody library

Anti-Cas9 nanobodies were developed in collaboration with Turkey Creek Biotechnology, Waverly, Tennessee, USA (in accordance with IACUC protocol 18–01) and the Vanderbilt Antibody and Protein Resource core facility (VAPR), Vanderbilt University, Nashville, Tennessee. We first immunized one alpaca “Flower Blossom” 8 times (at 2 week intervals) with purified *Streptococcus pyogenes* Cas9 (SpCas9) mixed 1:1 by volume in Gerbu adjuvant FAMA (item 3030 Gerbu F from GERBU Biotechnik). One week following the last immunization, blood was drawn into citrate containing blood bags, and PBMCs were isolated by centrifugation of ~100 mL of blood using SepMate centrifugal devices (STEMCELL Technologies). A cDNA library was made via reverse transcription using oligo dT primers and Superscript IV reverse transcriptase (ThermoScientific). A two-step, nested, PCR strategy was used to amplify coding regions of VHH fragments.^25,26^ The resulting PCR fragments were ligated into pBBR3, a modified pADL22 vector (Antibody Design Labs), containing sequences for C-terminal, HA and hexahistadine tags. The plasmids were electroporated into high-efficiency TG1 cells (Lucent), and phage were produced using CM13 helper phage.

A single round of panning was done against 10 μg SpCas9 immobilized in wells of a 96 well MaxiSorp plates. Three wells of a MaxiSorp plate were coated overnight with 10 μg of SpCas9 in PBS (phosphate buffered saline) with three PBS coated wells serving as controls. Wells were blocked with 2% nonfat milk in PBS, washed, and incubated with 2 × 10^11^ phage particles in blocking buffer for 1 hr. After extensive alternating washes with PBS and PBS + 0.5% Tween 20, phage were eluted with 100 μL 100 mM glycine pH 2.2, which was immediately neutralized with 100 μL 1 M Tris–HCl pH 8. Recovered phage were used to infect TG1 E. coli, and single clones were picked into deep well 96 blocks of terrific broth with 100 μg/mL ampicillin. Plates were grown at 37 °C for 5 hrs followed by 28 °C overnight. Bacteria were pelleted and lysed by two freeze–thaw cycles with a total of 400 μL of PBS pH 7.4. Positive clones were identified with a modified ELISA using a MaxiSorp plate coated with 1 μg SpCas9 per well and incubated with 25–50 μL of periplasmic extract. The plate was developed using an anti-HA (12CA5) antibody followed by HRP-labeled goat anti-mouse secondary (Jackson ImmunoResearch) and 1 Step Ultra-ELISA TMB substrate (ThermoFisher).

### Nanobody expression

Bacterial expression was done in T7 Shuffle *E. coli* (New England Bioloabs) with bacterial growth done at 37 °C in Luria Broth until OD600 reached 0.6–1.0, at which point 1 M IPTG was added to 1 mM and growth continued overnight at 18C. Eukaryotic expression was done Expi293 cells maintained in F17 Freestyle media (Thermo Fisher Scientific) and incubated at 37 °C and 8% CO_2_ with shaking. Cells were transfected using PEI^27^ at 2.5 × 10^6^ cells/mL with the nanobodies cloned into pcDNA3.4 (Thermo Fisher Scientific). These cultures were incubated at 37 °C and 8% CO_2_ with shaking for 7 days prior to being harvested.

### Nanobody purification

Purification from bacterial cultures involved clarification by centrifugation, and resuspension in phosphate buffered saline (25 mM sodium phosphate, 150 mM NaCl, pH 8, 1 μg/ml DNAse and 1 μg/ml lysozyme), followed by lysis in an Emulsiflex C3. The lysate was clarified by centrifugation, bound to Talon Resin, washed extensively with wash buffer (25 mM sodium phosphate, 150 mM NaCl, pH 8, 10 mM imidazole) and eluted with the same buffer supplemented with 200 mM imidazole.

Transfected Expi293F cells were harvested by centrifugation, filtered through a 0.22 μm membrane and loaded onto a 1 mL Complete His-tag Purification column. The column was then washed and eluted with an imidazole gradient from 10-200 mM in 25 mM sodium phosphate, 150 mM NaCl, pH 8. Fractions containing eluted nanobody were pooled.

### Cas expression and purification

Cas9 was expressed from Addgene plasmid 62,934 (pET-NLS-Cas9-6xHis), a gift from David Liu.^28^ Cas3 was expressed from Addgene plasmid 180,214 (pET28-T7-NlaCas3-NLS-6xHis), a gift from Yan Zhang.^29^ Cas12 was expressed from Addgene plasmid 113,430 (pMBP-AsCas12a), a gift from Jennifer Doudna.^30^ Cas14 was expressed from Addgen plasmid 112,506 (pLBH533_MBP-Cas14b2), a gift from Jennifer Doudna.^31^ All clones contained hexahistadine tags. Plasmids were transformed into BL21 Star (Thermo Fisher Scientific) with bacterial growth done at 37 °C in Luria Broth until OD600 reached ~1–1.2, at which point IPTG was added to 1 mM and growth continued overnight at 18 °C.

Bacteria were collected by centrifugation and resuspended in phosphate buffered saline (25 mM sodium phosphate, 150 mM NaCl, pH 8, 1 μg/ml DNAse and 1 μg/ml lysozyme), followed by lysis in an Emulsiflex C3. Lysates were clarified by centrifugation, the NaCl concentration was adjusted to 500 mM, and bound in batch to 2 mLs of Talon resin (Takara). The resin was poured into a column, washed with 100 mLs of 25 mM sodium phosphate, 500 mM NaCl, pH 7, and eluted with 25 mLs of 100 mLs of 25 mM sodium phosphate, 500 mM NaCl, 200 mM Imidazole, pH 7.5. For Cas9, all buffers also contained 10% glycerol.

### Chemical biotinylation of nanobodies

Chemical biotinylation with NHS-Biotin (Pierce) was done with freshly dissolved NHS-Biotin at a 1:20 M ratio (excess NHS-Biotin). Tris pH 8 was added to a final concentration of 10 mM to quench the biotinylation reaction. Biotinylation was verified by an enzyme-linked immunosorbent assay (ELISA) using streptavidin-conjugated horseradish peroxidase (HRP).

### Determination of relative SpCas9 binding affinity

ELISAs were performed to determine the relative binding affinities of our nanobodies for SpCas9. This assay involved incubating 1 μg of SpCas9 per well in 100 μL on a 96 well MaxiSorb plate overnight at 4 °C with shaking. The next day, the plate was washed, blocked with 2% BSA for two hours, and incubated with 100 μL of 3-fold serial dilutions of each biotinylated nanobody. The nanobodies were then detected with a streptavidin conjugated HRP. 75 μL Thermofisher One-Step Prep was then added to each well and quenched with 2 M sulfuric acid upon color change. A450 was then taken and plotted for data analysis using Prism.

The dose–response to a Cas9 ribonucleoprotein complex (RNP) was determined as above except using an RNP formed from Cas9 and a sgRNA targeted to the ROSA mT/mG plasmid, a gift from Liqun Luo (Addgene #17787).^20^ The activity of this RNP was verified prior to the ELISA by incubating 2× molar excess of RNP with linearized ROSA mT/mG plasmid DNA and detecting cleaved DNA via agarose gel electrophoresis.

### Determination of relative Cas3, Cas12, and Cas14 binding affinity

ELISAs were performed to determine is nanobodies C1, D9, or F9 cross-reacted with Cas3, Cas12, or Cas14. 1 μg per well of each Cas protein was incubated in 100 μL of PBS in a 96 well MaxiSorb plate overnight at 4 °C with shaking. The next day, the plate was washed, blocked with 2% BSA for two hours, and incubated with 100 μL of 3-fold serial dilutions of each biotinylated nanobody. The nanobodies were then detected with an anti-HA conjugated HRP (Roche). 75 μL Thermofisher One-Step Prep was then added to each well and quenched with 2 M sulfuric acid upon color change. A450 was then taken and plotted for data analysis using Prism.

### Epitope binning

Epitope binning was performed with a capture ELISA. A 96-well plate was first incubated overnight at 4 °C with 1 μg of unlabeled competing nanobody per well in 100 μL PBS. The plate was then washed and blocked for two hours at room temperature in 2% PBS-BSA. 100 μL of 100 ng/mL SpCas9 solution was then added to the plate and incubated for 2 h at room temperature. Biotinylated nanobodies were then added to the wells in a serial dilution series ranging from 300 ng/μL to 1.7 pg/μL, with the final volume being 100 μL for each well and incubated for one hour at room temperature. Streptavidin-HRP secondary antibody (100 μL of a 1:10,000 dilution) was then added to each well and incubated for 1 h at room temperature. of 1-Step Ultra TMB ELISA solution was added to each well (75 μL) and developed until there was a substantial color change in the plate, after which time 75 μL of 2 M Sulfuric Acid was then added to quench the solution and absorbance at 450 nm was recorded.

### Inhibition assay

For the inhibition assay, the spCas9 ribonucleoprotein complex (RNP) was first formed by incubating purified spCas9 with a sgRNA targeted to the ROSA mT/mG plasmid in a 1 to 1.25 M ratio for 30 min at 37 °C (Addgene #17787, a gift from Liqun Luo.^20^ A cleavage protocol was then performed with a 50 μL total volume containing 14 nM RNP, 28 nM nanobody, 1.4 nM linearized Rosa mT/mG plasmid, and 100 nM Magnesium in PBS. Each sample was incubated at 37 °C for one minute, before transferring 10 μL to a tube containing 2 μL 140 nM proteinase K and incubating this aliquot with the proteinase K for 15 min at 56 °C to stop the reaction. The remaining 40 μL of sample were then incubated for another 4 min, to bring the total time at 37 °C to 5 min, and 10 μL of sample was once again incubated with proteinase K to stop the reaction. This process was repeated until time points of 1 min, 5 min, 10 min and 1 h were achieved. The samples were then analyzed by agarose gel electrophoresis and the intensity of each band was quantified via Image J (https://doi.org/10.1038/nmeth.2089). Using these intensities, the percent DNA cleavage was determined for each sample.

### Mass photometry

The ability of nanobodies C1, F9, and D9 to bind SpCas9 simultaneously was evaluated by mass photometry (MP) using a Refeyn OneMP instrument (Refeyn Ltd).^32,33^ The instrument was calibrated using NativeMark Unstained Protein Standards (Thermo Fischer Scientific). Coverslips (Thorlabs) and gaskets (Grace Bio-Labs) were washed with 100% IPA then ddH_2_O 3 times and dried with dry nitrogen. 15 μl of PBS was added to the well to focus the instrument after which 5 μl of protein solution was added and mixed by pipetting and frame acquisition was started. The final concentration of SpCas9 was 25 nM and the final concentration of each nanobody was 50–100 nM. 6000 frames were acquired using AcquireMP (version 2.3.0; Refeyn Ltd) and standard settings. The frames were analyzed, and masses were estimated by fitting the data with Gaussian distribution using DiscoverMP (version 2.3.0; Refeyn Ltd).

## Supplementary Material

supplemental figure 3

supplemental figure 1

supplemental figure 2

supplemental figure legend

## Figures and Tables

**Table 1:** Gene usage of Cas9 nanobodies.

V/D/J Gene	# Clones	Percent
IGHV 3S66	26	51
IGHV 3S53	12	23.5
IGHV 3S65	11	21.6
IGHV 3S1	1	2
IGHV 3S61	1	2
IGHD 3	7	58.3
IGHD 5	2	16.7
IGHD 6	1	8.3
IGHD 2	1	8.3
IGHD 1	1	8.3
IGHJ 4	39	76.5
IGHJ 6	8	15.7
IGHJ 7	4	7.8

**Table 2:** V region mutations and amino acid changes from germline sequences.

Selected Clones	V-Gene Mutations	Amino Acid Changes	Non-CDR3 Lysines
C1	33	20	3
C7	37	21	5
F9	35	20	5
D9	14	7	5
G7	10	6	5
E6	8	5	5
F8	10	6	5
F1	7	3	5
G10	10	6	5
D5	41	20	5
F6	10	5	5
A6	44	23	5
G5	32	17	5
D8	12	6	5
E4	9	4	5

## Data Availability

Nanobody DNA sequences have been deposited in Genbank with accession numbers PP869893-PP869943.

## References

[1] Parums DV, (2024). Editorial: First regulatory approvals for CRISPR-Cas9 therapeutic gene editing for sickle cell disease and transfusion-dependent β-thalassemia e944204-1–4 Med. Sci. Monit. Int. Med. J. Exp. Clin. Res 30 10.12659/MSM.944204. PMC10913280 38425279

[2] Jinek M, Chylinski K, Fonfara I, Hauer M, Doudna JA, Charpentier E, (2012). A programmable dual-RNA-guided DNA endonuclease in adaptive bacterial immunity. Science 337, 816–821. 10.1126/science.1225829. 22745249 PMC6286148

[3] Gillmore JD, Maitland ML, Lebwohl D, (2021). CRISPR-Cas9 in vivo gene editing for transthyretin amyloidosis. Reply. N. Engl. J. Med 385, 1722–1723. 10.1056/NEJMc2114592. 34706181

[4] Frangoul H, Altshuler D, Cappellini MD, Chen Y-S, Domm J, Eustace BK, Foell J, de la Fuente J, Grupp S, Handgretinger R, Ho TW, Kattamis A, Kernytsky A, Lekstrom-Himes J, Li AM, Locatelli F, Mapara MY, de Montalembert M, Rondelli D, Sharma A, Sheth S, Soni S, Steinberg MH, Wall D, Yen A, Corbacioglu S, (2021). CRISPR-Cas9 gene editing for sickle cell disease and β-thalassemia. N. Engl. J. Med 384, 252–260. 10.1056/NEJMoa2031054. 33283989

[5] Cong L, Ran FA, Cox D, Lin S, Barretto R, Habib N, Hsu PD, Wu X, Jiang W, Marraffini LA, Zhang F, (2013). Multiplex genome engineering using CRISPR/Cas systems. Science 339, 819–823. 10.1126/science.1231143. 23287718 PMC3795411

[6] Komor AC, Kim YB, Packer MS, Zuris JA, Liu DR, (2016). Programmable editing of a target base in genomic DNA without double-stranded DNA cleavage. Nature 533, 420–424. 10.1038/nature17946. 27096365 PMC4873371

[7] Anzalone AV, Randolph PB, Davis JR, Sousa AA, Koblan LW, Levy JM, Chen PJ, Wilson C, Newby GA, Raguram A, Liu DR, (2019). Search-and-replace genome editing without double-strand breaks or donor DNA. Nature 576, 149–157. 10.1038/s41586-019-1711-4. 31634902 PMC6907074

[8] Rees HA, Liu DR, (2018). Base editing: precision chemistry on the genome and transcriptome of living cells. Nature Rev. Genet 19, 770–788. 10.1038/s41576-018-0059-1. 30323312 PMC6535181

[9] Klompe SE, Vo PLH, Halpin-Healy TS, Sternberg SH, (2019). Transposon-encoded CRISPR–Cas systems direct RNA-guided DNA integration. Nature 571, 219–225. 10.1038/s41586-019-1323-z. 31189177

[10] Strecker J, Ladha A, Gardner Z, Schmid-Burgk JL, Makarova KS, Koonin EV, Zhang F, (2019). RNA-guided DNA insertion with CRISPR-associated transposases. Science 365, 48–53. 10.1126/science.aax9181. 31171706 PMC6659118

[11] Guilinger JP, Thompson DB, Liu DR, (2014). Fusion of catalytically inactive Cas9 to FokI nuclease improves the specificity of genome modification. Nature Biotechnol. 32, 577–582. 10.1038/nbt.2909. 24770324 PMC4263420

[12] Knight SC, Tjian R, Doudna JA, (2018). Genomes in focus: development and applications of CRISPR-Cas9 imaging technologies. Angew. Chem. Int. Ed Engl 57, 4329–4337. 10.1002/anie.201709201. 29080263 PMC6014596

[13] Thakore PI, Black JB, Hilton IB, Gersbach CA, (2016). Editing the epigenome: technologies for programmable transcription and epigenetic modulation. Nature Methods 13, 127–137. 10.1038/nmeth.3733. 26820547 PMC4922638

[14] Qi LS, Larson MH, Gilbert LA, Doudna JA, Weissman JS, Arkin AP, Lim WA, (2013). Repurposing CRISPR as an RNA-guided platform for sequence-specific control of gene expression. Cell 152, 1173–1183. 10.1016/j.cell.2013.02.022. 23452860 PMC3664290

[15] Gilbert LA, Horlbeck MA, Adamson B, Villalta JE, Chen Y, Whitehead EH, Guimaraes C, Panning B, Ploegh HL, Bassik MC, Qi LS, Kampmann M, Weissman JS, (2014). Genome-scale CRISPR-mediated control of gene repression and activation. Cell 159, 647–661. 10.1016/j.cell.2014.09.029. 25307932 PMC4253859

[16] Jovčevska I, Muyldermans S, (2020). The therapeutic potential of nanobodies. BioDrugs 34, 11–26. 10.1007/s40259-019-00392-z. 31686399 PMC6985073

[17] Muyldermans S., (2013). Nanobodies: natural single-domain antibodies. Annu. Rev. Biochem 82, 775–797. 10.1146/annurev-biochem-063011-092449. 23495938

[18] Alamyar E, Giudicelli V, Li S, Duroux P, Lefranc M-P, (2012). IMGT/HIGHV-quest: The IMGT^®^ web portal for immunoglobulin (IG) or antibody and t cell receptor (TR) analysis from NGS high throughput and deep sequencing. Immunome Res. 08 10.4172/1745-7580.1000056.

[19] Kordus SL, Kroh HK, Rodríguez RC, Shrem RA, Peritore-Galve FC, Shupe JA, Wadzinski BE, Lacy DB, Spiller BW, (2023). Nanobodies against C. difficile TcdA and TcdB reveal unexpected neutralizing epitopes and provide a toolkit for toxin quantitation in vivo. PLOS Pathog. 19, e1011496. 37871122 10.1371/journal.ppat.1011496 PMC10621975

[20] Muzumdar MD, Tasic B, Miyamichi K, Li L, Luo L, (2007). A global double-fluorescent Cre reporter mouse. Genes N. Y. N 2000 (45), 593–605. 10.1002/dvg.20335. 17868096

[21] Tu Z, Huang X, Fu J, Hu N, Zheng W, Li Y, Zhang Y, (2020). Landscape of variable domain of heavy-chain-only antibody repertoire from alpaca. Immunology 161, 53–65. 10.1111/imm.13224. 32506493 PMC7450171

[22] Papikian A, Liu W, Gallego-Bartolomé J, Jacobsen SE, (2019). Site-specific manipulation of Arabidopsis loci using CRISPR-Cas9 SunTag systems. Nature Commun. 10, 729. 10.1038/s41467-019-08736-7. 30760722 PMC6374409

[23] Liu XS, Wu H, Ji X, Stelzer Y, Wu X, Czauderna S, Shu J, Dadon D, Young RA, Jaenisch R, (2016). Editing DNA methylation in the mammalian genome. Cell 167, 233–247.e17. 10.1016/j.cell.2016.08.056. 27662091 PMC5062609

[24] Hilton IB, D’Ippolito AM, Vockley CM, Thakore PI, Crawford GE, Reddy TE, Gersbach CA, (2015). Epigenome editing by a CRISPR/Cas9-based acetyltransferase activates genes from promoters and enhancers. Nature Biotechnol. 33, 510–517. 10.1038/nbt.3199. 25849900 PMC4430400

[25] Maass DR, Sepulveda J, Pernthaner A, Shoemaker CB, (2007). Alpaca (Lama pacos) as a convenient source of recombinant camelid heavy chain antibodies (VHHs). J. Immunol. Methods 324, 13–25. 10.1016/j.jim.2007.04.008. 17568607 PMC2014515

[26] Pardon E, Laeremans T, Triest S, Rasmussen SGF, Wohlkönig A, Ruf A, Muyldermans S, Hol WGJ, Kobilka BK, Steyaert J, (2014). A general protocol for the generation of Nanobodies for structural biology. Nature Protoc. 9, 674–693. 10.1038/nprot.2014.039. 24577359 PMC4297639

[27] Longo PA, Kavran JM, Kim M-S, Leahy DJ, (2013). Transient mammalian cell transfection with polyethylenimine (PEI). Methods Enzymol. 529, 227–240. 10.1016/B978-0-12-418687-3.00018-5. 24011049 PMC4012321

[28] Zuris JA, Thompson DB, Shu Y, Guilinger JP, Bessen JL, Hu JH, Maeder ML, Joung JK, Chen Z-Y, Liu DR, (2015). Cationic lipid-mediated delivery of proteins enables efficient protein-based genome editing in vitro and in vivo. Nature Biotechnol. 33, 73–80. 10.1038/nbt.3081. 25357182 PMC4289409

[29] R T, Rk K, Mj G, X Z, Y X, A K, Z H, Y Z, (2022). Cas11 enables genome engineering in human cells with compact CRISPR-Cas3 systems. Mol. Cell 82 10.1016/j.molcel.2021.12.032. PMC8964063 35051351

[30] Js C, E M, Lb H, M DC, X T, Jm P, Ja D, (2018). CRISPR-Cas12a target binding unleashes indiscriminate single-stranded DNase activity. Science 360 10.1126/science.aar6245. PMC6628903 29449511

[31] Harrington LB, Burstein D, Chen JS, Paez-Espino D, Ma E, Witte IP, Cofsky JC, Kyrpides NC, Banfield JF, Doudna JA, (2018). Programmed DNA destruction by miniature CRISPR-Cas14 enzymes. Science 362, 839–842. 10.1126/science.aav4294. 30337455 PMC6659742

[32] Young G, Hundt N, Cole D, Fineberg A, Andrecka J, Tyler A, Olerinyova A, Ansari A, Marklund EG, Collier MP, Chandler SA, Tkachenko O, Allen J, Crispin M, Billington N, Takagi Y, Sellers JR, Eichmann C, Selenko P, Frey L, Riek R, Galpin MR, Struwe WB, Benesch JLP, Kukura P, (2018). Quantitative mass imaging of single molecules. Science 360, 423. 10.1126/science.aar5839. 29700264 PMC6103225

[33] Ortega Arroyo J, Andrecka J, Spillane KM, Billington N, Takagi Y, Sellers JR, Kukura P, (2014). Label-free, all-optical detection, imaging, and tracking of a single protein. Nano Lett. 14, 2065–2070. 10.1021/nl500234t. 24597479 PMC4186656

