# Supplementary material for: A Nanobody Toolbox for Recognizing Distinct Epitopes on Cas9
Source: J Mol Biol. Author manuscript; Available in PMC 2025 Feb 25. (PMC11852565; doi:10.1016/j.jmb.2024.168836)
Supplement: supplemental figure 3 [file NIHMS2057313-supplement-supplemental_figure_3.docx]

**C.**

**B.**

**A.**
