# Supplementary material for: A Nanobody Toolbox for Recognizing Distinct Epitopes on Cas9
Source: J Mol Biol. Author manuscript; Available in PMC 2025 Feb 25. (PMC11852565; doi:10.1016/j.jmb.2024.168836)
Supplement: supplemental figure 1 [file NIHMS2057313-supplement-supplemental_figure_1.docx]

| Nanobody Name | Sequence (CDR1, CDR2, CDR3) |
| --- | --- |
| A10 | QVQLQESGGGLVQAGGSLRLSCAASGFTFDDYAIGWFRQAPGKEREGVSCISGSGGSPYYADSVKGRFTISSDNAKNTVYLQMNSLKPEDTAVYYCAADLWRLCQLDAEYDYWGQGTQVTVSS |
| A4 | QVQLQESGGGLVQAGGSLRLSCAASGFSFDDYAIGWFRQAPGKEREGVSCISPSDGSTDYADSVKGRFTISSDNAKNTVYLQMNSLKPEDTAVYYCAADQWRLCTSPINEYDYWGQGTQVTVSS |
| A6 | QVQLQESGGGAVQPGGSLGLSCTASGFNFETSTVGWFRQAPGKEYEGVSCINKGYEDTNYADSVKGRFTISRDAAKNTVYLQMDSLQPEDTATYYCAAHNEPYFCDYSGRFRWNEYSYYGQGTQVTVSS |
| A7 | QVQLQESGGGLVQTGGSLRLSCAASGFTLADYAIGWFRQAPGKERERVSCINSDGKSAHFPRVVKGRFTISRDNAKNTVYLQLNSLEPEDTAVYVCAADLKCGGQFAMRSADVSRFHSWGQGTQVTVSS |
| A8 | QVQLQESGGGLVQAGGSLRLSCVASENFSRDDTMGWYRQAPGKQREWVASITVVGTTNYADSVKGRFTISRDNPKNTHYLQMSSLKPEDTAVYYCYGRRRFRNYDYWGQGTQVTVSS |
| B2 | QVQLQESGGGLVQAGGSLRLSCAASGFSFDDYAIGWFRQAPGKEREGVSCISPSDGSTDYTDSVKGRFTISSDNAKNTVYLQMNSLKPEDTAVYYCAADQWRLCTSPINDYDYWGQGTQVTVSS |
| B4 | QVQLQESGGGLVQAGGSLRLSCAASGFTFDDYAIGWFRQAPGKEREGVSCISGSGGSPYYADSVKGRFTISSDNAKNMVYLQMNSLKPEDTAVYYCAADLWRLCQLDAEYDYWGQGTQVTVSS |
| B5 | QVQLQESGGGLVQAGGSLRLSCAASGFTFEDYLIGWFRQAPGKEREGVSCISSSDGSTYYADSVKGRFTISSDNAKNTVYLQMNSLKPEDTAVYYCAADLWSFCGSYYYSYEYDYWGQGTQVTVSS |
| B6 | QVQLQESGGALVQPGGSLRLSCAASGFSFSAYGMYWVRQAPGKGLEWVSFIAGVTTNYADSVKGRFTISRDNAKNTLYLQMNSLKSEDTAVYYCAKNHGTIWQGFDGMDYWGKGTQVTVSS |
| B7 | QVQLQESGGGLVQAGGSLRLSCAASGFTFDDYAIGWFRQAPGKGREGVSCIGSSGGSPYYADSVKGRFTISSDNAKNTVYLQMNSLKPEDTAVYYCAADLWRLCQLDAEYDYWGQGTQVTVSS |
| C1 | QVQLQESGGGLVQAGGSLRLSCAASGTSSRLHPMSWYRQIPGNQRQLVARITSTGSTNYGDSVQDRFTISRDNGKNTIYLQMNSLKPEDTAVYYCRIVAEGRLYWGEGTQVTVSS |
| C10 | QVQLQESGGGLVQPGGSLRLSCVASENFSRDDTVGWYRQAPGKQREWVASITVVGTTNYADSVKGRFTISRDNPKNTHYLQMSSLKPEDTAVYYCYGRRRFRNYDYWGQGTQVTVSS |
| C3 | QVQLQESGGGLVQPGGSLRLSCAASGFTLDYYAIGWFRQAPGKEREGVSCISSSGGSTNYADSVKGRFTISRDNAKNTVYLQMTSLKPEDTAVYYCAAWEGSSEYCPLQFSADFASWGRGTQVTVSS |
| C4 | QVQLQESGGGLVRAGGSLRLSCAASGFTFDDYAIGWFRQAPGKEREGVSCISSSAGSTYYADAVKGRFTISSDNAKNTVYLQMNSLKPEDTAVYYCAAEKWSLCGSYYLRGGMDYWGKGTQVTVSS |
| C7 | QVQLQESGGGLVQAGGSLRLSCVASENFSRDDTVGWYRQAPGKQREWVASITVVGTTNYADSVKGRFTISRDNPKNTHYLQMSSLKPEDTAVYYCYGRRRFRNYDYWGQGTQVTVSS |
| C8 | QVQLQESGGGLVQPGGSLRLSCAASGFTFDDYAIGWFRQAPGREREGVSCISTSAGATAYADSVKGRFTISSDNAKNTVYLQMNSLKPEDTAVYYCAADQWRLCTSPINDYDYWGQGTQVTVSS |
| D10 | QVQLQESGGGLVQAGGSLRLSCAASGFTFDDYAIGWFRQAPGKEREGVSCIASSGGSTDYADSVKGRFTISSDNAKNTVYLQMNSLKPEDTAVYYCAADQWRLCTSPINDYDYWGQGTQVTVSS |
| D11 | QVQLQESGGGLVQAGGSLRLSCAASGFTFDDYAIGWFRQAPGKEREGVSCISSSAESTYYADSVKGRFTISSDNAKNTVYLQMNSLKPEDTAVYYCAAEKWILCGSYYLRGGMDYWGKGTQVTVSS |
| D2 | QVQLQESGGGLVQSGESLRLSCATSGFNLDNYAIAWFRQAPGKEREGVACSSSDGSSDGSTYYADSVKGRFTISSDNAKNTVYLQMNSLKPEDTAVYYCAADLWRLCQLDAEYDYWGQGTQVTVSS |
| D3 | QVQLQESGGGLVQPGGSLRLSCAASGFILDFYAIGWFRQAPGKEREGVSCISSSGGRTNYADSVKGRFTISRDNAKNTVYLQMNSLKPEDTAVYSCAAWEGSTRYCPIQTSADFVSWGQGTQVTVSS |
| D5 | QVQLQESGGGEVQAGGSLRLSCAASGVSFSIDTVNWYRQAPGKRRVWVGGITAGGLTNYPASVKGRFTISRDNAKNTVYLEMNSLKFEDTAVYYCNIRDYWGQGTQVTVSS |
| D6 | QVQLQESGGGLVQAGGSLRLSCAASGFTFDDYAIGWFRQAPGKERERVSCIGSSDGSTYYADSVKGRFTISSDNAKNTVYLQMNSLKPEDTAVYYCAADLWRLCQLDAEYDYWGQGTQVTVSS |
| D7 | QVQLQESGGGLVQAGGSLRLSCAASGFTFDDYAIGWFRQAPGKEREGVSCIASSGGSTDYADSVKGRFTISSDNAKNTVYLQMNSLKPEDTAVYYCAADQWRLCTSPINDYDYWGQGTQVTVSS |
| D8 | QVQLQESGGGLVQPGGSLRLSCAASGFILDFYAIGWFRQAPGKEREGVSCISSSGGRTNYADSVKGRFTISRDNAKNTVYLQMNSLKPEDTAVYSCAAWEGSTRYCPIQTSADFVSWGQGTQVTVSS |
| D9 | QVQLQESGGGLVQAGGSLRLSCAASGFSFDDYAIGWFRQAPGKEREGVSCISPSDGSTDYTDSVKGRFTISSDNAKNTVYLQMNSLKPEDTAVYYCAADQWRLCTSPINDYDYWGQGTQVTVSS |
| E10 | QVQLQESGGGLVQPGGSLRLSCAASGFILDFYAIGWFRQAPGKEREGVSCISSSGGRTNYADSVKGRFTISRDNAKNTVYLQMNSLKPEDTAVYSCAAWEGSTRYCPIQTSADFVSWGQGTQVTVSS |
| E3 | QVQLQESGGGLVQAGGSLRLSCVASENFSRDDTVGWYRQAPGKQREWVASITVVGTTNYADSVKGRFTISRDNPKNTHYLQMSSLKPEDTAVYYCYGRRRFRNYDYWGQGTQVTVSS |
| E4 | QVQLQESGGGLVQPGGSLRLSCAASGFTLDYYAIGWFRQAPGKEREGVSCISSSGGSTNYADSVKGRFTISRDNAKNTVYLQMTGLKPEDTAVYYCAAWEGSSEYCPLQFSADFASWGRGTQVTVSS |
| E5 | QVQLQESGGGLVQAGGSLRLSCAASGFTFDDYAIGWFRQAPGKEREGVSCIGSSDGSTYYADSVKGRFTISSDNAKNTVYLQMNSLKPEDTAVYYCAADLWRLCQLDAEYDYWGQGTQVTVSS |
| E6 | QVQLQESGGGLVQAGGSLRLSCAASGFTFDDYAIGWFRQAPGKERERVSCIGSSDGSTYYADSVKGRFTISSDNAKNTVYLQMNSLKPEDTAVYYCAADLWRLCQLDAEYDYWGQGTQVTVSS |
| E8 | QVQLQESGGGLVQAGGSLRLSCAASGNIPSFGYMGWYRQAPGKQREMVALIGSGGTKNYADSVKGRFTISMDNAKKSVYLQMNSLRPDDTAVYYCKLFMGAAYWGQGTQVTVSS |
| F1 | QVQLQESGGGLVQAGGSLRLSCAASGFTFDDYAIGWFRQAPGKEREGVSCISSSDGSTYYADSVKGRFTISSDNAKNTVYLQMNSLKPEDTAVYYCAAELYVLCGAGYPPNEYDYWGQGTQVTVSS |
| F3 | QVQLQESGGGLVQAGGSLRLSCVASENFSRDDTVGWYRQAPGKQREWVASITVVGTTNYADSVKGRFTISRDNPKNTHYLQMSSLKPEDTAVYYCYGRRRFRNYDYWGQGTQVTVSS |
| F4 | QVQLQESGGGLVQAGGSLRLSCAASGFTFDDYAIGWFRQAPGKEREGVSCIGGSDASTYYADSVKGRFTISSDNAENTVYLQMNSLKPEDTAVYYCAADLWRLCQLDAEYDYWGQGTQVTVSS |
| F5 | QVQLQLLDRSFPRLLQESGGGLVQAGGSLRLSCAASESIFRMELMEWYRQAPGKQRELVATITRSGSTNYSDSVKGRFIISSDNAKNSVYLQMNSLKAEDTAVYLCHARTWTSYWGQGTQVTVSS |
| F6 | QVQLQESGGGLVQAGGSLRLSCAASGFTFEDYLIGWFRQAPGKEREGVSCISSSDGSTYYADSVKGRFTISSDNAKNTVYLQMNSLKPEDTAVYYCAADLWSFCGSYYYSYEYDYWGQGTQVTVSS |
| F7 | QVQLQESGGGLVQAGGSLRLSCAASGFTFDDYAIGWFRQAPGKERERVSCIGSSDGSTYYADSMKGRFTISSDNAKNTVYLQMNSLKPEDTAVYYCAADLWRLCQLDAEYDYWGQGTQVTVSS |
| F8 | QVQLQESGGGLVQAGGSLRLSCAASGFTFDDYAIGWFRQAPGKEREGVSCISGSGGSPYYADSVKGRFTISSDNAKNTVYLQMNSLKPEDTAVYYCAADLWRLCQLDAEYDYWGQGTQVTVSS |
| F9 | QVQLQESGGGLVQAGGSLRLSCVASENFSRDDTVGWYRQAPGKQREWVASITVVGTTNYADSVKGRFTISRDNAKNTHYLQMSSLKPEDTAVYYCYGRRRFRDYDYWGQGTQVTVSS |
| G10 | QVQLQESGGGLVQAGGSLRLSCAASGFTFDDYIIAWFRQAPGKEREGVSCISSSDGSTYYVDSVKGRFTISSDNAKNTVYLQMNSLKPEDTAVYYCAADLWSFCGSHYYSYEYDYWGQGTQVTVSS |
| G3 | QVQLQESGGGLVQPGGSLRLSCAASGSTLDYYAIGWFRQAPGKEREGVLCISSSGGITYYIDSVKGRFTISRDNAKNTVYLQMNSLKPEDTGVYYCATEGIECNEDEYDFWGQGTQVTVSS |
| G4 | QVQLQESGGGLVQAGGSLRLSCVASENFSRDDTVGWYRQAPGKQREWVASITVVGTTNYADSVKGRFTISRDNAKNTHYLQMSSLKPEDTAVYYCYGRRRFRDYDYWGQGTQVTVSS |
| G5 | QVQLQESGGGLVQPGGSLRLSCAASGFTLADYAIGWFRQAPGKERERVSCINSDGKSAHFPRVVKGRFTISRDNAKNTVYLQLNSLEPEDTAVYVCAADLKCGGQFAMRSADVSRFHSWGQGTQVTVSS |
| G7 | QVQLQESGGGLVQAGGSLRLSCAASGFTFDDYAIGWFRQAPGKEREGVSCIASSGGSTDYADSVKGRFTISSDNAKNTVYLQMNSLKPEDTAVYYCAADQWRLCTSPINDYDYWGQGTQVTVSS |
| G9 | QVQLQESGGGLVEPGGSLRLSCAASGFTLADYAIGWFRQAPGKERERVSCINSDGKSAHFPRVVKGRFTISRDNAKNTVYLQLNSLEPEDTAVYVCAADLKCGGQFAMRSADVSRFHSWGQGTQVTVSS |
| H1 | QVQLQESGGGLVQAGGSLRLSCAASGFSFDDYAIGWFRQVPGKEREGVSCISPSDGSTDYTDSVKGRFTISSDNAKNTVYLQMNSLKPEDTAVYYCAADQWRLCTSPINDYDYWGQGTQVTVSS |
| H2 | QVQLQESGGGLVQPGGSLRLSCAASGFTFDDYAIGWFRQAPGKEREGVSCISPSDGSTDYADSVKGRFTISSDNAKNTVDLQMNSLKPEDTAVYYCAADQWRLCTSPINDYDYWGQGTQVTVSS |
| H4 | QVQLQESGGGLVQAGGSLRLSCAASGFTFDDYAIGWFRQAPGKEREGVSCISSSGGSPYYADSVKGRFTISSDNAKNTVYLQMNSLKPEDTAVYYCAADLWRLCQLDAEYDYWGQGTQVTVSS |
| H5 | QVQLQESGGGAVQPGGSLGLSCTASGFNFETSTVGWFRQAPGKEYEGVSCINKGYEDTNYADSVKGRFTISRDAAKNTVYLQMDSLQPEDTATYYCAAHNEPYFCDYSGRFRWNEYSYYGQGTQVTVSS |
| H6 | QVQLQESGGGLVQAGGSLRLSCVASENFSRDDTVGWYRQAPGKQREWVASITVVGTTNYADSVKGRFTISRDNPKNTHYLQMSSLKPEDTAVYYCYGRRRFRNYDYWGQGTQVTVSS |
| H7 | QVQLQESGGGLVQPGGSLRLSCAASGFTFDDYAIGWFRQAPGKEREGVSCISGSGGRPYYADSVKGRFTISSDNAKNTVYLQMNSLKPEDTAVYYCAADLWRLCQLDAEYDYWGQGTQVTVSS |
