# Supplementary material for: A Nanobody Toolbox for Recognizing Distinct Epitopes on Cas9
Source: J Mol Biol. Author manuscript; Available in PMC 2025 Feb 25. (PMC11852565; doi:10.1016/j.jmb.2024.168836)
Supplement: supplemental figure legend [file NIHMS2057313-supplement-supplemental_figure_legend.docx]

Supplemental Figure 1. Sequences in the anti-Cas9 Nanobody Library. The corresponding DNA sequences have been deposited in Genbank. Accession numbers PP869893-PP869943.

Supplemental Figure 2. Agarose gel electrophoresis used to measure percent DNA cleaved in figure 4D. (A) Samples after 1 minute at 37 °C. (B) Samples after 5 minutes at 37 °C. (C) Samples after 10 minutes at 37 °C. (D) Samples after 1 hour at 37 °C.

Supplemental Figure 3. Interactions between C1, D9, and F9 and Cas proteins. (A) Dose response of C1 to Cas9, Cas3, Cas12, Cas14, and BSA. (B) Dose response of D9 to Cas9, Cas3, Cas12, Cas14, and BSA (C) Dose response of F9 to Cas9, Cas3, Cas12, Cas14, and BSA Error bars are SEM of triplicate measurements.
